# The natural product, echinatin, protects mice from methicillin-resistant *Staphylococcus aureus* pneumonia by inhibition of alpha-hemolysin expression

**DOI:** 10.3389/fmicb.2023.1128144

**Published:** 2023-04-14

**Authors:** Wei Zhang, Qing Gong, Zhitong Tang, Xin Ma, Zhuoer Wang, Jiyu Guan, Li Wang, Yicheng Zhao, Ming Yan

**Affiliations:** ^1^The Third Affiliated Hospital of Changchun University of Chinese Medicine, Changchun, China; ^2^The Affiliated Hospital of Changchun University of Chinese Medicine, Changchun, China; ^3^Jilin Provincial Cancer Hospital, Changchun, China; ^4^Changchun University of Chinese Medicine, Changchun, China; ^5^Key Laboratory of Zoonosis, Ministry of Education, College of Veterinary Medicine, Jilin University, Changchun, China; ^6^College of Integrated Traditional Chinese and Western Medicine, Changchun University of Chinese Medicine, Changchun, China

**Keywords:** antivirulence, α-hemolysin, methicillin-resistant *Staphylococcus aureus*, pneumonia, echinatin

## Abstract

Antimicrobial resistance (AMR) is a global, multifaceted crisis that poses significant challenges to the successful eradication of devastating pathogens, particularly methicillin-resistant *Staphylococcus aureus* (MRSA), a persistent superbug that causes devastating infections. The scarcity of new antibacterial drugs is obvious, and antivirulence strategies that reduce the pathogenicity of bacteria by weakening their virulence have become the subject of intense investigation. Alpha-hemolysin (Hla), a cytolytic pore-forming toxin, has a pivotal role in *S. aureus* pathogenesis. Here, we demonstrated that echinatin, a natural compound isolated from *licorice*, effectively inhibited the hemolytic activity of MRSA at 32 μg/mL. In addition, echinatin did not interfere with bacterial growth and had no significant cytotoxicity at the inhibitory concentration of *S. aureus* hemolysis. Heptamer formation tightly correlated with Hla-mediated cell invasion, whereas echinatin did not affect deoxycholic acid-induced oligomerization of Hla. Echinatin affected hemolytic activity through indirect binding to Hla as confirmed by the neutralization assay and cellular thermal shift assay (CETSA). Furthermore, qRT–PCR and western blot analyses revealed that echinatin suppressed Hla expression at both the mRNA and protein levels as well as the transcript levels of Agr quorum-sensing system-related genes. Additionally, when echinatin was added to a coculture system of A549 cells and *S. aureus*, it significantly reduced cell damage. Importantly, echinatin exhibited a significant therapeutic effect in an MRSA-induced mouse pneumonia model. In conclusion, the present findings demonstrated that echinatin significantly inhibits the hemolysin effect and may be a potential candidate compound for combating drug-resistant MRSA infections.

## Introduction

Antibiotic resistance is a worldwide threat. Pathogenic bacteria have evolved multiple strategies to combat antibiotics, including blocking antibiotic access to targets, mutating antibiotic targets, biochemically modifying targets, and directly modifying antibiotics to acquire resistance (Walsh, 2000). Currently, there are few effective ways to circumvent resistance mechanisms.

*Staphylococcus aureus* (*S. aureus*), a multidrug-resistant bacterium, has become an urgent concern. Since methicillin-resistant *Staphylococcus aureus* (MRSA) first appeared in the 1960s, it has become endemic in hospitals and health care facilities worldwide, causing persistent damage to the economy and human health (Parker et al., 2016). *S. aureus*, a member of ESKAPE, has developed resistance to many first-line drugs in ESKAPE infections compared to other antibiotic-sensitive bacteria (Cohen et al., 2018; Mulani et al., 2019). *S. aureus* is associated with skin and soft tissue infections (SSTIs) as well as life-threatening sepsis and pneumonia, and MRSA is a pathogen of particular concern in intensive care units (ICUs) (Turner et al., 2019). Identifying viable strategies to combat multidrug-resistant *S. aureus* is a challenge for scientists and clinicians.

Fortunately, effective antimicrobial techniques are being developed with antivirulence targeting becoming more prevalent. Antivirulence therapeutic agents bypass the mechanisms of antibiotic resistance and eliminate or reduce the options for resistance (Buroni and Chiarelli, 2020). Additionally, unlike conventional antibiotics, which can kill or limit bacterial growth, antivirulence medications target the virulence components found in bacteria and aim to disarm pathogens (Heras et al., 2015). As a result, virulence factors provide a wide range of intriguing targets for novel therapies (Casadevall and Pirofski, 1999).

The pathogenicity of *S. aureus* is dependent on a variety of virulence factors, including cell surface proteins, polysaccharides, and secreted toxins. The latter causes tissue damage, promotes bacterial dissemination, promotes metastatic growth in distant organs, and allows the pathogen to evade host innate immunity (Rooijakkers et al., 2005; Nizet, 2007; Adhikari et al., 2012). Pore-causing alpha-hemolysin (Hla), also known as a-toxin (AT), is a pore-forming exotoxin produced by almost all virulent strains of the bacterium. The lethal properties of Hla include hemolytic activity, cytotoxicity, dermonecrosis, and other endotoxins (Wächter et al., 2021). Hla-mediated cell death is caused by the formation of a transmembrane pore in the plasma membrane of susceptible cells, allowing leakage of intracellular material, and it has been associated with several *S. aureus* diseases, including SSTI (Kennedy et al., 2010) and pneumonia (Inoshima et al., 2011). Given that Hla plays a key role in the pathogenesis of *S. aureus* infection (Caiazza and O'Toole, 2003; Bubeck Wardenburg and Schneewind, 2008; Kennedy et al., 2010), this toxin is a prime candidate target against MRSA and the current target of choice for the development of *S. aureu*s vaccines.

Natural products are crucial in the development of medical therapeutic approaches due to their low toxicity, structural diversity, and significant biological activity (Deng et al., 2020). Approximately 70% of all anti-infective drugs have been derived from natural products found in the environment. For this reason, we screened Hla inhibitors in a library of natural product compounds (Newman and Cragg, 2020). Fortunately, echinatin, a flavanol compound, exhibited better inhibition against MRSA and MSSA and clinical isolates of *S. aureus* at low concentrations. Echinatin is isolated from the herb licorice, which has also been reported to have hepatoprotective and anti-inflammatory effects (Liang et al., 2018).

Herein, the analysis of echinatin inhibitory effects against Hla of *S. aureus* revealed the mechanism of action. More importantly, the therapeutic effect of echinatin was preliminarily evaluated at the cellular level and *in vivo* level by establishing a MRSA-induced lethal pneumonia infection model in mice. The present study provides experimental and theoretical evidence for the natural product, echinatin, in anti-MRSA infection and provides new candidate compounds to combat multidrug-resistant *S. aureus* infections.

## Materials and methods

### Bacterial strains, reagents, and growth conditions

MRSA USA 300 (BAA-1717) and methicillin-sensitive *S. aureus* Newman (ATCC 8325) were purchased from American Type Culture Collection (ATCC). Laboratory-preserved human lung adenocarcinoma cells (A549 cells) were used to evaluate the cytotoxicity of the compounds. The *S. aureus* Hla-deficient mutant (Δ*Hla*) and SA1B3G clinical MRSA isolate were kept in our laboratory. *E. coli* and *S. aureus* were grown in Luria-Bertani (LB) broth and trypticase broth (TSB), respectively, placed at 37°C and shaken at 220 rpm/min. Cells were cultured in 1640 medium with 10% fetal bovine serum, 100 U/mL penicillin, and 100 mug/mL streptomycin. Echinatin, a natural product, was obtained from Pufei De Biotech Co., Ltd. (Chengdu, China) at 98% purity. The quality test report of echinatin is presented in Supplementary Figure 1. Other routine reagents were obtained from Sangon Biotech (Shanghai, China).

### Construction, expression, and purification of the Hla protein

To produce the recombinant Hla protein, the pET28a-hla prokaryotic expression vector was generated. In brief, the hla gene was amplified from the genome of *S. aureus* USA300 *via* PCR using the primers listed in Table 1. The PCR product was double-digested with BamHI and XhoI, and the product was then ligated into pET28a using T4 DNA ligase (Takara, Dalian, China). The recombinant plasmids were then transformed into *E. coli* DH5α by the heat shock method. After verification by sequencing, the plasmids were transformed into *E. coli* BL21 (DE3) for subsequent expression. *E. coli* containing recombinant plasmids were cultured to an OD_600_ of 0.8–1.0 with the addition of isopropyl-beta-D-thiogalactoside (IPTG, 0.5 mM), and incubation was continued at 16°C until an OD_600_ of 2.5 was reached. Bacteria were then collected by centrifugation and lysed by sonication, and the supernatant was collected. Hla protein was purified using the HIS-Selected Nickel Affinity Gel system (Beyotime, Shanghai, China).

**Table 1:** Primers used in this study.

Primer name	Sequences (5′ – 3′)
*Hla*-F	CGCGGATCCGCAGATTCTGATATTAATATTAAAAC
*Hla*-R	CCGCTCGAGTTAATTTGTCATTTCTTCTTTTTC
rt-*16sRNA*-F	TGGAGCATGTGGTTTAATTCGA
rt-*16sRNA*-R	TGCGGGACTTAACCCAACA
rt-*hla*-F	ACAATTTTAGAGAGCCCAACTGAT
rt-*hla*-R	TCCCCAATTTTGATTCACCAT
rt-*agrA*-F	GCAGTAATTCAGTGTATGTTCA
rt-*agrA*-R	TATGGCGATTGACGACAA
rt-*pvl*-F	GAGGTGGCCTTTCCAATACAAT
rt-*pvl*-R	CCTCCTGTTGATGGACCACTATTA
rt-*RNAIII*-F	TTCACTGTGTCGATAATCCA
rt-*RNAIII*-R	GGAAGGAGTGATTTCAATGG
rt-*psmα*-F	TATCAAAAGCTTAATCGAACAATTC
rt-*psmα*-R	CCCCTTCAAATAAGATGTTCATATC

### Screening for Hla inhibitors

The compounds (64 μg/mL) were added to the culture of *S. aureus* USA300 at an initial OD_600_ of 0.3, and the incubation was continued until an OD_600_ of 2.5 was reached. The supernatant of the above cultures (100 μL) was mixed with defibrinated rabbit blood (25 μL) and PBS to 1 mL. Triton X-100 and *S. aureus* USA300 were used as negative controls for hemolytic activity, and PBS and myricetin (Wang et al., 2020) were used as positive controls. The mixture was incubated at 37°C for 1 h before being centrifuged to collect the supernatant, and the absorbance value at 543 nm was determined (Duan et al., 2018).

### Minimum inhibition concentration (MIC)

In accordance with the protocol described in the Clinical Laboratory Standards Association 2017 standards (El-Shaer et al., 2017), a multiplicative dilution method was employed to determine the MIC of echinatin against *S. aureus* USA300. Overnight cultures of *S. aureus* USA300 were inoculated into CAMHB medium at a ratio of 1:1000 and incubated until the logarithmic growth phase. In a 96-well plate, 100 μL of *S. aureus* USA300 containing 1 × 10^5^ CFU was added to each well, and16-512 μg/mL echinatin was then added to the broth. The control group consisted of medium only. After incubating the plate at 37°C for 16 h, the absorbance at 600 nm was measured with a microplate reader (Thermo Fisher, United States).

### Growth assay

Growth curves were utilized to determine if echinatin affects the growth of USA300. *S. aureus* USA300 was resuspended in TSB at a ratio of 1:100 and incubated for overnight. After adjusting the *S. aureus* culture to an OD_600_ of 0.1 with fresh TSB media, 64 μg/mL echinatin or an equal volume of DMSO was added. The OD_600_ values in each group of bacterial solutions were measured at 1-h intervals for 24 h using an UV spectrophotometer. Based on these values, the growth curve of *S. aureus* USA300 was plotted.

### Hemolytic assay

The inhibitory effect of echinatin on the hemolysis of *S. aureus* Newman, *S. aureus* USA300, and the SA1B3G clinical isolate was assessed by detecting the destruction of red blood cells. *S. aureus* cultures with an initial OD_600_ of 0.3 were treated with various concentrations of echinatin (4 to 32 μg/mL), and incubation was continued until the OD_600_ was 2.5. The supernatant of the above cultures (100 μL) was mixed with prewashed (in PBS) defibrinated rabbit blood (25 μL) and PBS to 1 mL. Under the condition that other components remained constant, Triton X-100 and *S. aureus* culture supernatant were employed as negative controls. The mixture was incubated at 37°C for 1 h before being centrifuged to collect the supernatant, and the absorbance value at 543 nm was determined.

### Neutralization activity

*Staphylococcus aureus* USA300 overnight culture was diluted 1:100 into fresh medium and incubated until the OD_600_ reached 2.5. The culture supernatant was collected and saved. The following system was contained in a 1.5 mL EP tube: 25 μL of RBCs, 100 μL of culture supernatant, 10 μL of echinatin at a final concentration (4 to 32 μL/mL) and 865 μL of PBS. Following an hour of incubation at 37°C, 100 μL of supernatant was added to a 96-well plate by low-speed centrifugation and photographed, and the absorbance value at 543 nm was measured.

### Heptamer formation

To examine the assembled heptameric state, we tested the impact of echinatin on Hla oligomerization as previously described (Wang et al., 2016, 2020). Purified Hla protein (0.5 mg/mL) was incubated with different concentrations of echinatin (0 to 64 μg/mL) at 22°C for 25 min. Then, the samples were incubated at 55°C for 10 min with protein loading buffer without β-mercaptoethanol. Subsequently, each group of samples was subjected to 8% SDS–PAGE followed by western blot analysis.

### Cellular thermal shift assay (CETSA)

The CETSA is based on a temperature-dependent thermal transfer assay using bacterial lysates as described previously (Ren et al., 2022). The bacterial lysates were divided equally into two portions. One portion was treated with echinatin (64 μg/mL), and the other portion was treated with DMSO. The lysates were then placed in the dark at 37°C for 1 h. Subsequently, the supernatant was collected after centrifugation, transferred to PCR tubes, incubated for 5 min at a specific temperature for Hla protein, and immediately cooled on ice for 3 min. The samples were mixed with protein loading buffer and then subjected to 10% SDS–PAGE. The band intensity, which is correlated with protein stability, was quantified using ImageJ software (National Institute of Health, Bethesda, MD, United States).

### Quantitative real-time PCR (qRT–PCR)

*Staphylococcus aureus* USA300 culture at an initial OD_600_ of 0.3 was treated with echinatin (0–32 μg/mL) or DMSO, and the culture was incubated while shaking until an OD_600_ of 2.5 was reached. The bacteria were subsequently collected, and total RNA was extracted using TRIzol reagent (Tiangen, Beijing, China) according to the manufacturer’s instructions. Total RNA (1 μg) was transcribed into complementary DNA (cDNA) using the HiScript^®^ II 1st Strand cDNA Synthesis Kit (#R211, Vazyme, Naijing, China). Gene-specific primers were designed based on the base sequences provided by NCBI (Table 1). qRT–PCR was performed using an ABI 7900HT Real-time PCR system in combination with qPCR SYBR Green premix (Vazyme, Naijing, China) and the following cycle parameters: 95°C for 30 s; and 40 cycles of 95°C for 5 s, 60°C for 30 s and 72°C for 30 s. The transcription levels, which were normalized to *the 16S RNA*, were calculated using the 2^−ΔΔCT^ method.

### Western blot

*Staphylococcus aureus* cultures were treated with various echinatin concentrations (0 to 32 μg/mL) and incubated with shaking until the late logarithmic growth period was reached. The supernatant was then collected and mixed with protein loading buffer for 10% SDS–PAGE. Proteins were then transferred onto PVDF membranes by a semidry transfer device. The membranes were blocked with 5% bovine serum albumin (BSA) for 2 h at room temperature and then washed three times with PBST. The membrane was then incubated with the primary rabbit anti-Hla antibody (1:3000, Sigma–Aldrich) followed by incubation with the HRP-labeled goat anti-rabbit IgG (1:5000, Sigma–Aldrich) secondary antibody. After washing the membrane three times, the bands were observed by a chemiluminescence detection device after adding BeyoECL Star reagent (Beyotime, Beijing, China).

### Infection of A549 cells and lactate dehydrogenase (LDH) assay

A549 cells were inoculated into 24-well plates (2 × 10^4^ cells/well) and placed in a cell culture incubator for 24 h. The medium was removed, and fresh 1,640 medium (supplemented with 10% serum) containing *S. aureus* USA300 suspension (MOI = 50, 50 μL) and different concentrations of echinatin (0–32 μg/mL) was added to the cell culture plates followed by incubation for 6 h (Wang et al., 2020). For the live-dead cell assays, calcein AM/PI dye was used to determine the survival of A549 cells *via* fluorescence microscopy. Untreated cells were used as the blank control group.

For the LDH assays, the plates were incubated for 6, 12, or 24 h. A549 cells without any treatment were used as the control group. The supernatant was collected, and an LDH assay was performed to assess the protective effect of echinatin on A549 cells infected with *S. aureus* by measuring the release of the cell membrane enzyme LDH from damaged cells. The LDH activity present in the culture medium was determined using an LDH kit (Beyotime, Beijing, China).

### MTT assay

The toxicity of echinatin in A549 cells was assessed by an MTT assay kit (Beyotime, Beijing, China). A549 cells were seeded into a 96-well cell culture plate at a density of 5 × 10^3^ cells per well. After treatment with echinatin (0–32 μg/mL) for 24 h, the cells were washed with PBS, and MTT solution (10 μL of 5 mg/mL) was added to each well followed by incubation for an additional 4 h. Then, 100 μL of DMSO was added to dissolve the crystals, and the absorbance was analyzed using a 96-well plate reader at a wavelength of 490 nm.

### MRSA-infected pneumonia murine model

Seven-week-old C57BL/6J mice (approximately 22 g) were selected to establish a mouse pneumonia infection model induced by MRSA USA300. Mice were provided by Liaoning Changsheng Biotechnology Co. (Changchun, China), given adequate water and food, and acclimatized and fed for 1 week. Before the formal experiment, the *S. aureus* suspension was prepared. Overnight-grown *S. aureus* USA300 was transferred to fresh TSB medium at a 1:100 ratio and incubated overnight at 37°C. Bacteria were collected by low-speed centrifugation and resuspended in PBS for intranasal lung infections. The mice were randomly divided into 4 groups: the *S. aureus* USA300-infected group (WT), echinatin-treated group, uninfected control group, and Δ*Hla*-infected group (*n* = 24).

*Staphylococcus aureus* USA300 (2 × 10^8^ CFU) was given to each mouse for a survival study. The treatment group (*n* = 10) was given 60 mg/kg echinatin to mice subcutaneously within 1 h after infection with *S. aureus*; the infected group was given equal amounts of normal saline (0.05% DMSO) in the same way. The mice were then readministered *via* subcutaneous injection every 12 h, and the survival was monitored at 12 h intervals until 96 h.

For analysis of bacterial load and pulmonary pathological changes, mice were infected by intranasal injection of USA300 (1 × 10^8^ CFU). Mice were grouped (*n* = 8) and treated in the same manner as the survival analysis experiments. After 48 h of infection, the mice were euthanized. Right lung tissue was collected under aseptic conditions, weighed, and homogenized, and then the homogenate was appropriately diluted, spread on TSA agar plates and placed in an incubator for colony counting the next day. The left lung was collected, photographed, and subsequently placed in formalin and further stained with H&E after routine processing, such as paraffin embedding and sectioning, for observation by light microscopy. The remaining left lung lobe was also used to detect changes in the wet to dry weight ratio.

In addition, the amount of cytokines in the alveolar lavage fluid of mice was measured (*n* = 6). The amount and mode of administration in mice were consistent with the bacterial load analysis. After 48 h of treatment, bronchoalveolar lavage fluid studies were performed by injecting 1 mL of sterile PBS into the trachea and collecting the lavage fluid, and the kit assessed the concentration of cytokines (IL-6, #PI326; TNF-α, #PT512; IL-1β, #PI301, Beyotime, China).

### Flow cytometry

Mice were given 60 mg/kg echinatin by subcutaneous injection within 1 h after infection with a nonlethal dose of *S. aureus* USA300 (1 × 10^8^ CFU). Mice were euthanized after 24 h, and lung tissue cells were collected. Subsequently, erythrocytes were removed with ACK lysis buffer (R20172, Shanghai Yuanye Bio-Technology Co., Ltd), and the cells were resuspended in FACS buffer (PBS containing 2% fetal bovine serum and 1 mM EDTA). The following antibodies were used for FACS analysis: Ly6-G (FITC conjugated, clone 1A8) and CD11b (PE conjugated, clone M1/70). Cell suspensions were mixed with antibodies and incubated on ice for 30 min. After washing twice with FCS, cells were collected and resuspended in FCS, and samples were analyzed using flow cytometry.

### Statistical analysis

Data are expressed as the mean ± SD, and *p* ≤ 0.05 was considered statistically significant. Data were analyzed using two-tailed t tests, Mann–Whitney tests, and analysis of variance (ANOVA) followed by a log-rank test as appropriate. All statistical analyses were performed using GraphPad Prism version 8.0.

## Results

### Echinatin does not affect the growth of *Staphylococcus aureus*

**Figure 1:**
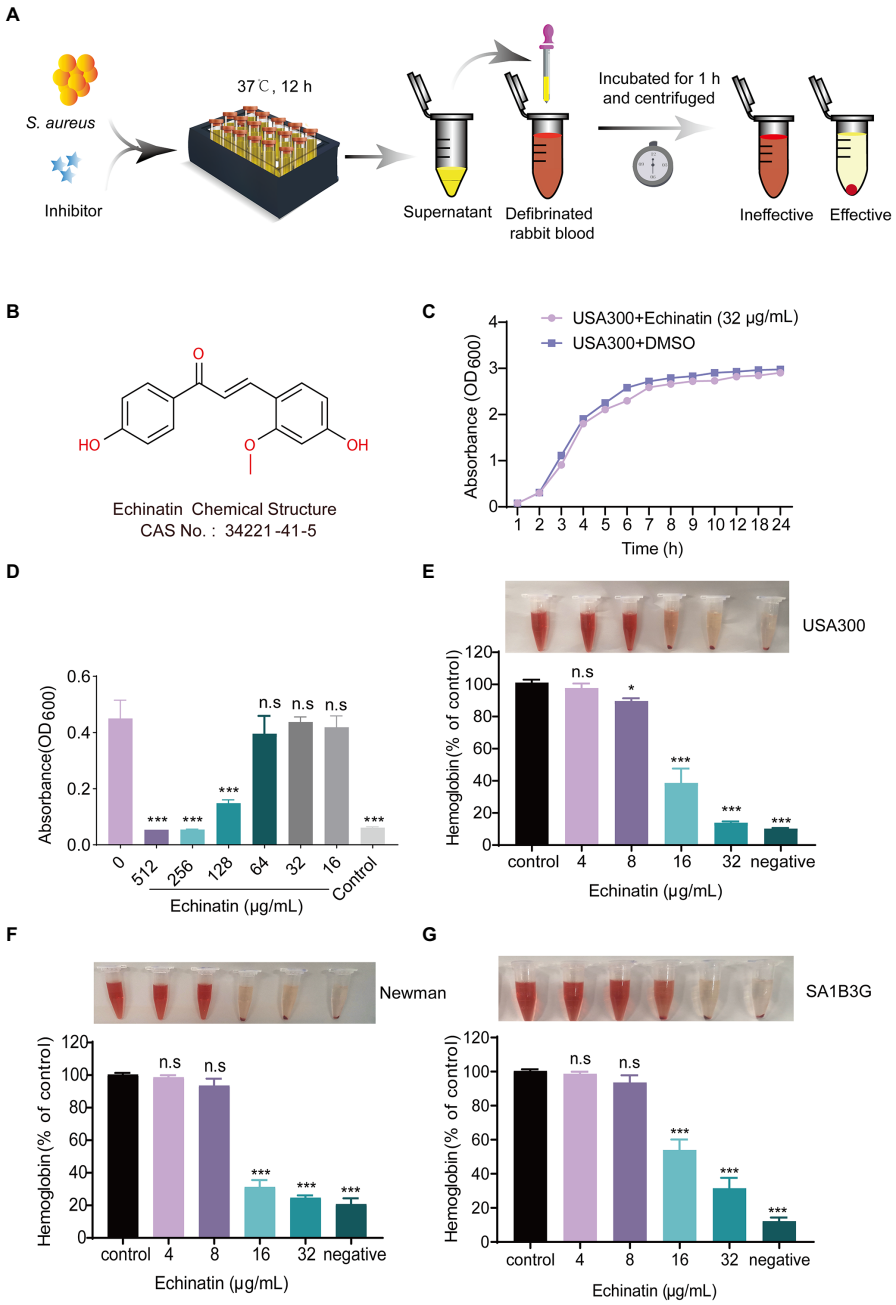
Echinatin inhibits the hemolysis of *Staphylococcus aureus* without affecting bacterial growth. **(A)** *In vitro* manual screening system for Hla inhibitors of *S. aureus* based on defibrinated rabbit blood. **(B)** Chemical structure of echinatin. **(C)** Growth rate of *S. aureus* USA300 grown in TSB at 37°C with 32 μg/mL echinatin or DMSO. **(D)** The bacterial inhibitory ability was determined by the MIC of echinatin against USA300 by the microbroth dilution method. **(E–G)** The effects of echinatin on the hemolytic activity of *S. aureus* USA300, Newman, and SA1B3G *in vitro*. Triton X-100 was used as a negative control. Data are presented as the mean ± SD, and the experiments were performed in triplicate. ^*^p < 0.05, ^**^p < 0.05, and ^***^p < 0.001.

Considering the multiple biological activities of flavonoids, we screened for Hla inhibitors in a library containing 55 flavonoids, and the screening process is shown in Figure 1A. According to the initial screening, three of the compounds (trifolirhizin, echinatin, and isobacachalcone) inhibited the hemolytic activity of *S. aureus* USA300 at the initial drug concentration (64 μg/mL) with a hit rate of approximately 5.45%. After removing low MIC and insignificant effects (Figure 1B), echinatin was selected as the candidate for further investigations (Supplementary Table 1).

Unlike the direct bactericidal effect of antibiotics, antivirulence drugs aim to reduce the production of virulence factors without interfering with bacterial growth. For this purpose, we tested whether echinatin affects the growth kinetics of *S. aureus* USA300 at different concentrations and monitored their growth over time. As shown in Figure 1C, there was no difference in the growth rate between the 32 μg/mL echinatin-treated group and DMSO-treated group. Moreover, the MIC of echinatin against USA300 was determined to be 256 μg/mL by the microbroth dilution method, which indicated that echinatin had no significant growth inhibitory effect on MRSA USA300 (Figure 1D).

### Echinatin inhibits the hemolytic ability of *Staphylococcus aureus*

Echinatin suppressed the Hla activity of *S. aureus* USA300 in a dose-dependent manner. Compared to the control, the hemolytic activity was significantly decreased to 13.92% when echinatin was added at a concentration of 32 μg/mL (*p* < 0.01) (Figure 1E). We next investigated the inhibitory effect of echinatin on MSSA Newman Hla activity, and the hemolytic activity was 24.74% after treatment with 32 μg/mL echinatin (Figure 1F). In addition, echinatin suppressed hemolysis in a dose-dependent manner in clinical isolates of *S. aureus* SA1B3G (Figure 1G). These findings demonstrated that echinatin at lower doses suppresses the hemolytic activity of clinical isolates and *S. aureus*.

### Echinatin does not affect heptamer formation in *Staphylococcus aureus*

**Figure 2:**
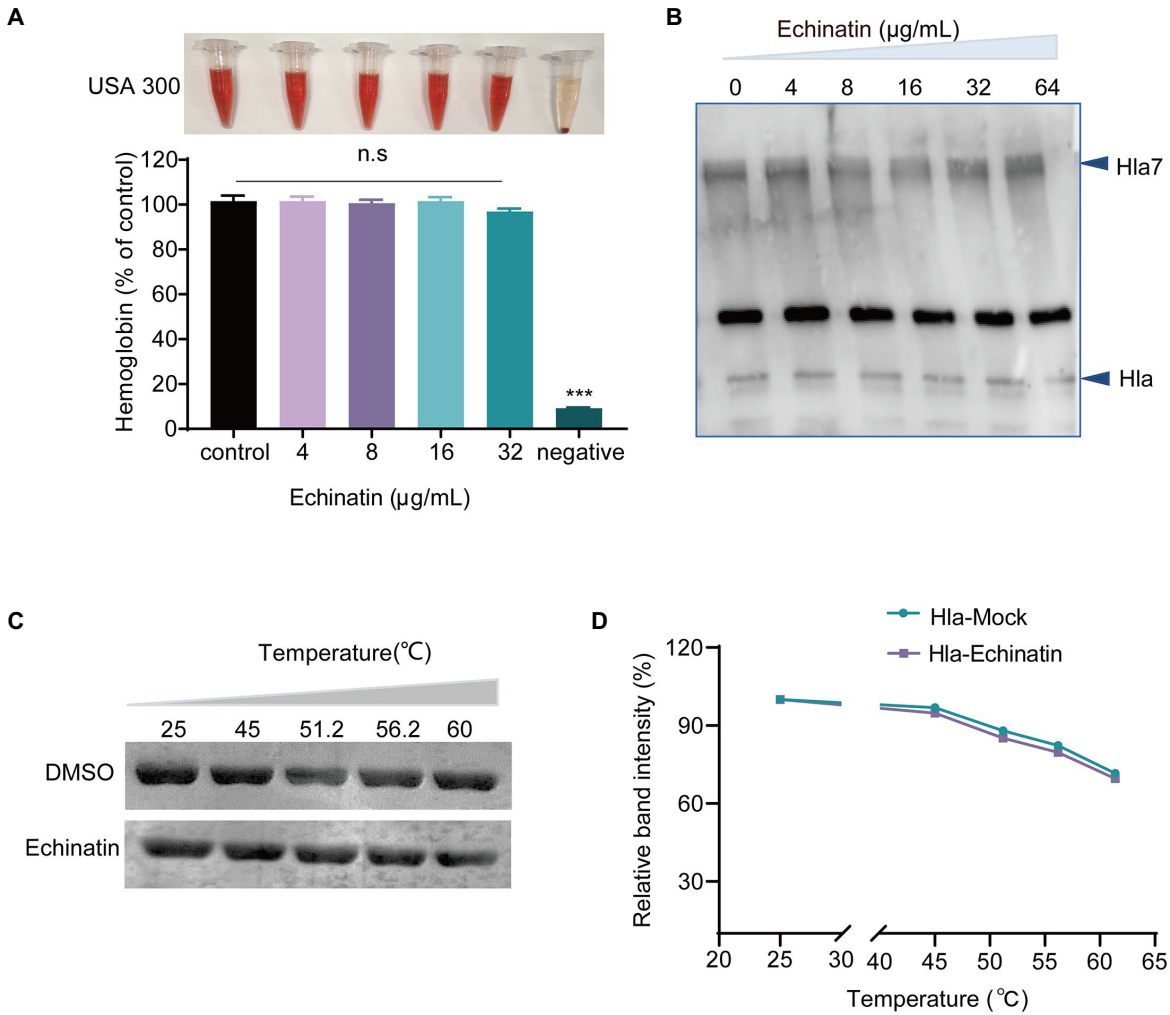
Echinatin does not affect heptamer formation in *S. aureus*. **(A)** The supernatant of *S. aureus* USA300 culture and echinatin was added to PBS containing defibrinated rabbit blood to assess the neutralizing effect of echinatin on Hla. **(B)** Effect of various concentrations of echinatin on oligomers of Hla. The formation of Hla heptamers was determined by western blot analysis after treatment with 5 mM deoxycholic acid in the presence or absence of echinatin. **(C,D)** CETSA was performed to determine the direct interaction between echinatin and Hla. Echinatin or DMSO was incubated with Hla at the indicated temperatures (25, 45, 51.2, 56.2, and 60°C). Subsequently, SDS–PAGE was performed, and ImageJ was used for data analysis. Data are presented as the mean ± SD (*n* = 3). ^***^p < 0.001.

To further elucidate the mechanism by which echinatin inhibits hemolysis, a neutralizing activity assay was performed. The hemolysis assay showed that echinatin had no neutralizing activity against Hla in the supernatant of *S. aureus* (Figure 2A). In addition, wild-type Hla is secreted as a water-soluble monomer outside of *S. aureu*s and self-assembles into a homopolymeric heptamer with a transmembrane structural domain (Wei et al., 2018). To this end, we further investigated whether echinatin inhibits hemolysis by affecting Hla polymerization. With increasing echinatin concentrations, heptamer formation was not significantly affected (Figure 2B), which suggested that echinatin had no effect on Hla heptamer formation. Furthermore, CETSA is a method to detect direct drug-target interactions by quantifying changes in the thermal stability of proteins after ligand binding in intact cells (Asawa et al., 2020). The CETSA demonstrated that there was no significant difference between the echinatin-treated group and the DMSO-treated group as the temperature increased (*p* > 0.001), which implied that there was no direct interaction between echinatin and Hla (Figures 2C,D). Thus, these findings suggested that the inhibitory effect of echinatin on *S. aureus* hemolysis activity is not achieved through direct binding to Hla.

### Echinatin inhibits *Hla*, *RNA III,* and *agrA* transcription

**Figure 3:**
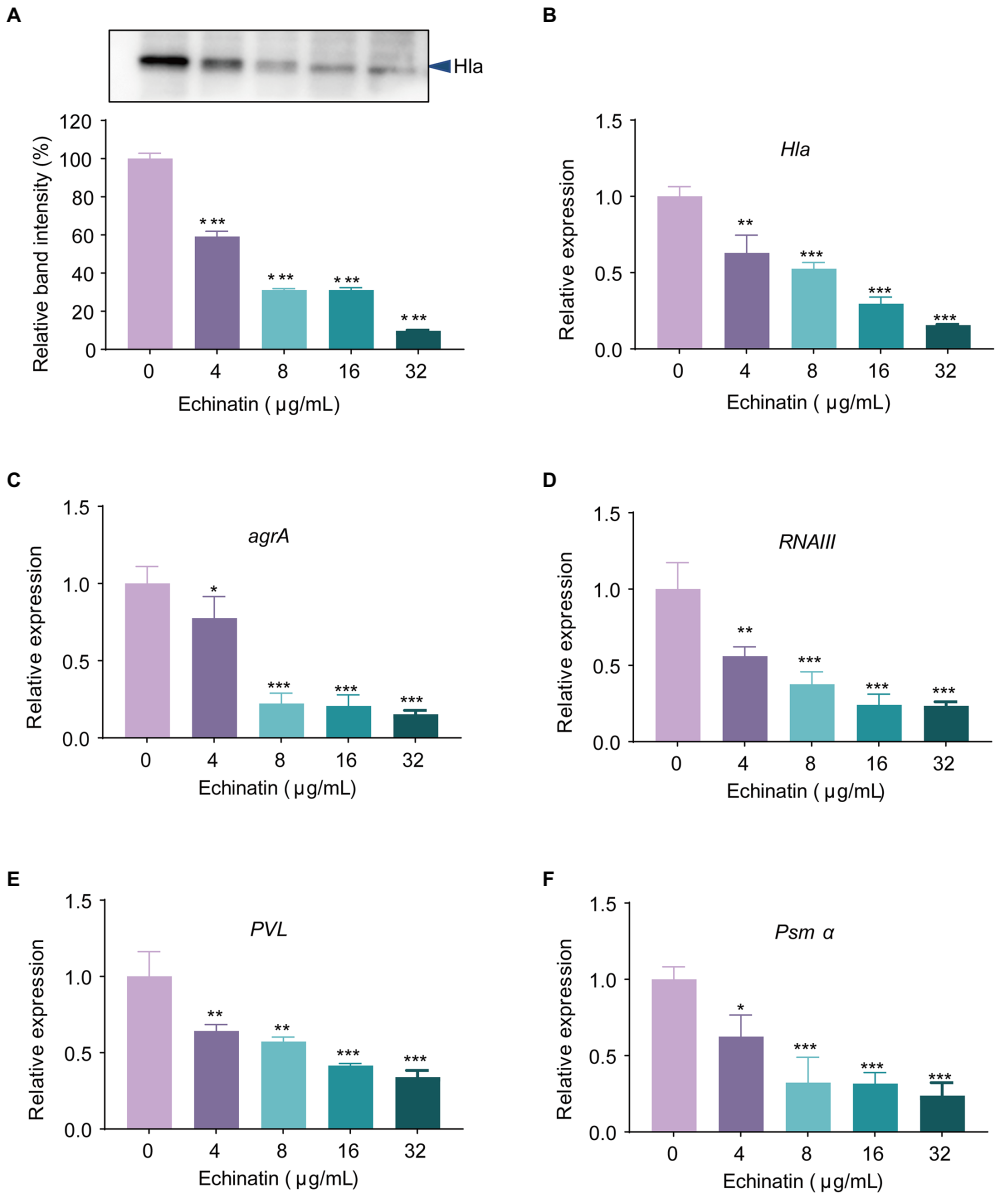
Echinatin significantly decreases the expression of Hla and Agr system regulatory-related genes. **(A)** Expression levels of Hla in *S. aureus* USA300 after treatment with different concentrations of echinatin (0 to 32 μg/mL) were determined by western blot analysis. Transcription levels of **(B)** *hla*, **(C)** *agrA*, **(D)** *RNAIII*, **(E)** *pvl*, and **(F)** *psmα* were determined in *S. aureus* USA300 treated or untreated with echinatin, and *16S RNA* was used as the internal reference gene. Experiments were performed in triplicate. *p < 0.05, **p < 0.01, and ***p < 0.001 compared to WT.

Because echinatin does not bind to Hla to inhibit hemolysis, we next investigated whether echinatin inhibits hemolysis by reducing Hla levels using qRT–PCR and western blot analyses. The expression level of Hla protein was significantly decreased by echinatin in a concentration-dependent manner (Figure 3A; *p* < 0.001). The Hla expression level after treatment with echinatin at 32 μg/ml was only 9.43% of that in the WT group. qRT-PCR analysis indicated that treatment with echinatin (32 μg/mL) significantly decreased Hla mRNA levels by 7-fold compared to the WT group (Figure 3B). The quorum sensing system is an important factor in the regulation of Hla, and once the threshold level of activated AgrA is reached, it binds to the P3 promoter and affects the *RNAIII* transcription factor, which in turn triggers the expression of a range of toxins and virulence factors, including Hla and Panton-Valentine leucocidin (PVL) (Greenberg et al., 2018). In addition, phosphorylation of AgrA directly regulates phenol-soluble modulin (PSMα) expression by binding to the promoter region of the PSMα operon and activating transcription (Queck et al., 2008).

Subsequently, we further examined the effect of echinatin on the transcriptional levels of the upstream regulatory genes, namely, *agrA* and *RNAIII*. As shown in Figures 3C,D, echinatin treatment at 32 μg/mL significantly inhibited the transcription of *agrA* and *RNAIII* by 7-fold and 5-fold, respectively, compared to the WT group (*p* < 0.001). In addition, PVL and PSMα are virulence factors governed by the Agr system and are closely associated with the pathogenicity of *S. aureus*. As expected, the transcript levels of *pvl* and *psmα* were differentially reduced in echinatin-treated *S. aureus* and displayed a dose dependence (Figures 3E,F). Collectively, these findings revealed that echinatin influences the production of Hla by affecting the Agr system.

### Protective effects of echinatin against *Staphylococcus aureus*-mediated A549 cell injury

**Figure 4:**
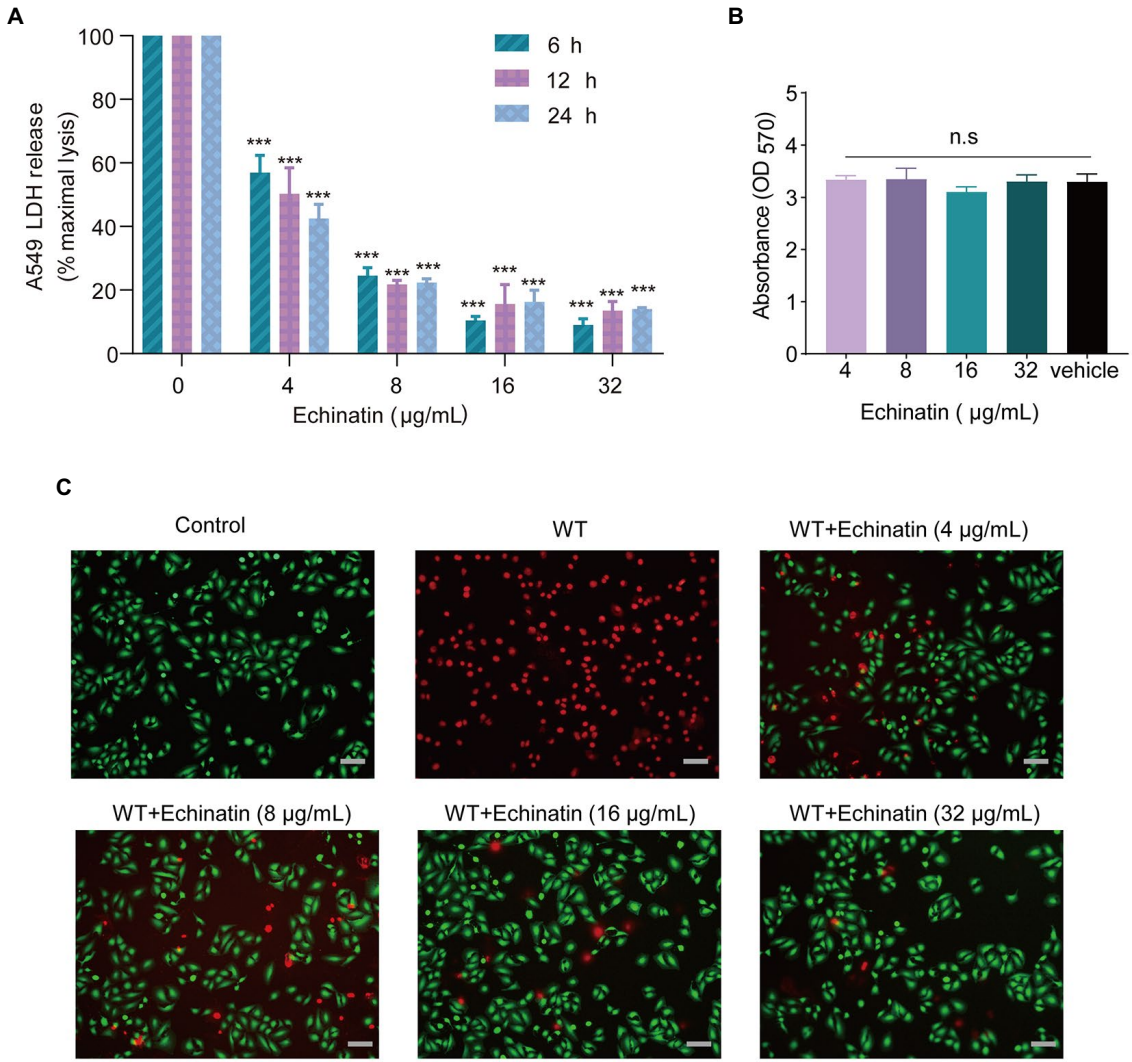
Protective effects of echinatin against *S. aureus*-mediated A549 cell injury. **(A)** After 24 h of incubation with the indicated concentrations of echinatin, the cell viability of A549 cells was determined using the MTT assay. **(B)** Different concentrations of echinatin-treated *S. aureus*-infected A549 cells and an LDH release assay were used to assess cell survival. **(C)** Echinatin attenuated *S. aureus* Hla-mediated damage to A549 cells. Live (green)/dead (red) agents were used to detect the survival of A549 cells after *S. aureus* infection with or without echinatin treatment, and images were acquired by fluorescence microscopy (Scale bar = 50 μm). Experiments were performed in triplicate. ^*^p < 0.05, ^**^p < 0.01, and ^***^p < 0.001 compared to the control.

Calcein AM/PI staining indicated that the *S. aureus*-treated A549 cells had high levels of cell death. However, echinatin showed a strong protective effect, even at doses as low as 4 μg/mL, as evidenced by a reduction in the proportion of PI-positive cells (Figure 4A).

A549 cells were infected with *S. aureus* USA300 and treated with echinatin, and cell survival was then assessed by an LDH assay to transiently mimic the therapeutic effect of echinatin after MRSA invasion. The results showed that echinatin significantly reduced the release of LDH compared to that in the USA300-infected group (*p* < 0.05), which indicated that echinatin reduced the damage caused to A549 cells by MRSA (Figure 4B). Subsequently, the IC_50_ of LDH release was further calculated to be 4.501, 4.278, and 4.122 μg/mL at 6, 12, and 24 h, respectively. There was no significant difference in the IC_50_ values with time, indicating that echinatin can act quickly and maintain a stable effect for a certain period of time. These findings demonstrated that echinatin, even at low concentrations, significantly reduced LDH release at 6, 12, and 24 h. Together, these results confirmed our hypothesis that echinatin protects A549 cells from *S. aureus*-induced damage.

In addition, MTT studies revealed that echinatin did not affect the viability of A549 cells at doses that reduced hemolytic activity, showing that echinatin is relatively safe and has no cytotoxicity (Figure 4C).

### Echinatin protects mice from *Staphylococcus aureus* pneumonia infection and reduces lung inflammation

**Figure 5:**
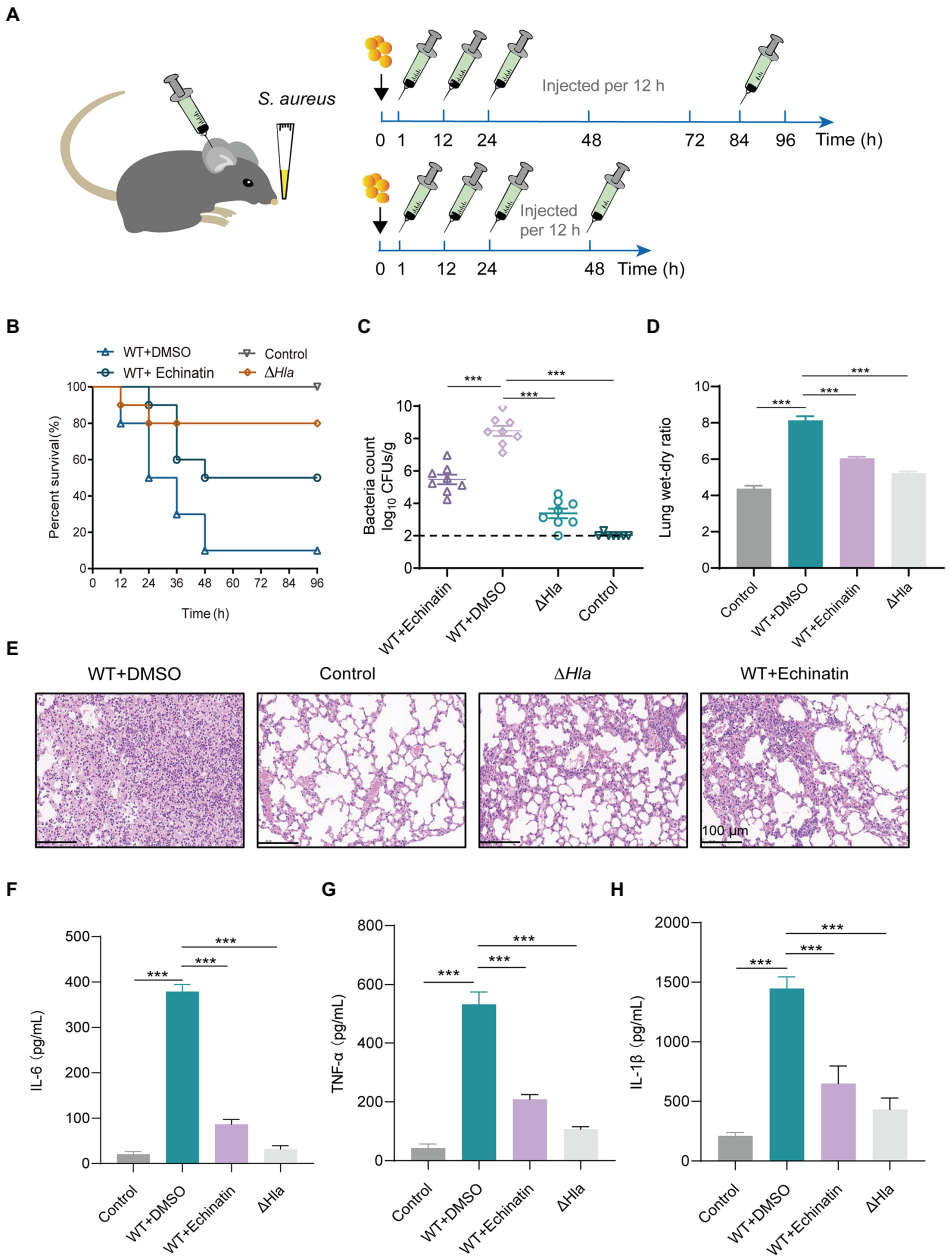
Echinatin protects against *S. aureus* pneumonia infection in mice. **(A)** Flow chart for the establishment of an *S. aureus*-induced pneumonia infection model in mice. *S. aureus* USA300 (2 × 10^8^ or 1 × 10^8^ CFU/mouse) or PBS was used to infect C57BL/6J mice *via* nasal drops, and mice were then treated with echinatin (60 mg/kg, i.h.) or DMSO (as a vehicle) to assess the therapeutic effect on *S. aureus*-induced pneumonia. **(B)** The survival rate of mice in each group was monitored at 12 h intervals for 96 h (*n* = 10). **(C)** Bacterial burden in the lung was determined after treatment with or without 60 mg/kg echinatin (*n* = 8). **(D)** Measurement of wet/dry weight ratios in the lungs of *S. aureus*-infected mice after echinatin treatment revealed the extent of lung damage. **(E)** Histopathological analysis of mouse lungs harvested after infection with *S. aureus* in the presence or absence of echinatin. Scale bar = 100 μm. **(F–H)** The levels of TNF-α, IL-1β, and IL-6 in bronchoalveolar lavage fluid (BALF) were determined by ELISAs. The data are presented as the mean ± SD. ^*^p < 0.05, ^**^p < 0.05, and ^***^p < 0.001.

Given that *S. aureus* USA300 has been linked to severe respiratory infections, we sought to determine the efficacy of echinatin against pneumonia caused by *S. aureus* USA300. First, we established a mouse pneumonia infection model by nasal drip injection of an *S. aureus* suspension. As shown in Figure 5A, echinatin was subcutaneously injected 1 h after infection and at 12 h intervals, and the survival was examined within 96 h. The survival rate of mice within 96 h of infection was only 10%, but an obvious increase in the survival rate from 10 to 50% was observed after treatment with echinatin (Figure 5B). These data indicated that echinatin significantly increases the survival rate of mice with *S. aureus* pneumonia.

Subsequently, the number of bacteria in the mouse lung tissue of each group was further assessed. After echinatin treatment, the amount of bacteria recovered from mouse lung homogenates infected with wild-type *S. aureus* was significantly reduced from 8.47 ± 0.87 log_10_ CFUs/g to 5.47 ± 0.85 log_10_ CFUs/g (*p* < 0.001) (Figure 5C).

Additionally, to measure the severity of lung damage in mice, lung tissues were collected, and the fresh tissues were weighed. The tissues were then dried in a medium temperature chamber at 60°C for 48 h, and the dried tissues were weighed. A significant loss in the wet/dry lung weight ratio was observed after echinatin treatment (Figure 5D).

Regarding pathological changes in mice, there was a greater infiltration of inflammatory cells into the lungs of the infected group and a significant reduction in the accumulation of inflammatory cells in the alveolar space after treatment with echinatin (Figure 5E).

We next examined the changes in the levels of several typical inflammation-related factors in the bronchoalveolar lavage fluid of each group. IL-1β, IL-6, and TNF-α were decreased by varying degrees in the bronchoalveolar lavage fluid of the echinatin-treated infected mice (Figures 5F–H).

Neutrophils and macrophages play a key role in the invasion of pathogenic bacteria in organisms and phagocytic clearance of pathogenic bacteria. The degree of inflammation in mice was further analyzed based on the number of neutrophils and macrophages. The population of immune cells in the lungs of uninfected mice was relatively low, comprising approximately 1.94% of the total lung cell population (Supplementary Figure 2A). In the early stage of infection, *S. aureus* released a large amount of toxins and other exoproteins, which caused a large influx of immune cells into the lungs, and the number of neutrophils and macrophages in the lung tissue of mice was relatively high, accounting for approximately 49.4% of total lung cells (Supplementary Figure 2B). In contrast, both neutrophils and macrophages in the lungs were significantly reduced by 14.6% in the echinatin-treated group compared to the infected group, indicating that echinatin was effective in relieving inflammation (Supplementary Figure 2C). These results suggested that echinatin effectively alleviates inflammation in the lungs of mice and reduces the aggregation of neutrophils and macrophages in the lungs of *S. aureus*-infected mice.

## Discussion

MRSA is a major cause of community- and hospital-acquired infections worldwide. In recent years, new anti-MRSA drugs, such as vancomycin, daptomycin, ceftaroline (Lodise and Low, 2012) and linezolid (Liu et al., 2021), have been clinically applied. These newly marketed antibiotics are mostly based on the structural optimization and modification of existing antibiotics without new breakthroughs in antibacterial mechanisms. The consequent tendency of bacteria to develop cross-resistance has led to a much shorter life cycle of antibacterial drugs. The search for alternative strategies has been a global pressing public health problem.

*Staphylococcus aureus* expresses many virulence factors, including various toxins and exoproteins, which weaken the innate immune system by directly lysing phagocytes or inactivating key innate response molecules as well as by causing tissue destruction and metastatic growth of bacteria to other tissues and organs to damage the host (Adhikari et al., 2012). Targeting *S. aureus* virulence-associated factors is an effective way to control MRSA. Hla induces alterations in ion gradients across the host cell membrane, disrupts the integrity of the cell membrane, and activates stress signaling pathways, leading to cell death. Furthermore, *S. aureus* uses Hla to avoid macrophage killing *via* NLRP3-dependent effects on mitochondrial trafficking (Cohen et al., 2018). Hla allows *S. aureus* to breach the host immune barrier and cause severe infections, including sepsis, pneumonia (McElroy et al., 1999; Bubeck Wardenburg et al., 2007), and severe skin infections. To combat *S. aureus* infection, monoclonal antibodies (mAbs) targeting Hla have been created, such as MEDI4893, which disrupts hemolytic activity by preventing heptamer formation and interfering with interactions with ADAM10 (Ragle and Bubeck Wardenburg, 2009; Foletti et al., 2013; Oganesyan et al., 2014). Given the critical role of Hla in the pathogenesis of *S. aureus*, it has emerged as an attractive therapeutic target in the fight against *S. aureus*.

In view of the obvious advantages of natural products in terms of structural novelty, biocompatibility, and functional diversity, we proposed to screen small molecule compounds from the natural product library that can effectively inhibit hemolysis. Among them, echinatin inhibited the hemolytic activity of several strains, including MRSA, MSSA, and clinical isolates, and no significant bacterial inhibition or cytotoxicity was observed at this concentration. These results suggested that echinatin is promising for further investigation.

Hla binds to the ADAM10 receptor on the cell membrane surface and forms heptamers, which in turn rupture the cell membrane (Inoshima et al., 2011; Seilie and Bubeck Wardenburg, 2017). Thus, affecting Hla heptamer formation influences hemolytic activity. Several natural products, such as myricetin (Wang et al., 2020), baicalin (Qiu et al., 2012) and curcumin (Wang et al., 2016), have been reported to interfere with the formation of Hla heptamers, thereby inhibiting hemolysis. To this end, we first confirmed that echinatin inhibits hemolysis in a manner that does not occur through direct binding to Hla by a neutralization assay, CETSA, and heptamer assay. However, the mechanism of action needs to be further explored.

In addition to directly affecting the formation of Hla heptamers, interference with upstream systems and thus regulation of hemolysin expression can also interfere with hemolysis. The expression of *S. aureus* Hla is controlled by a cascade regulatory network that has been relatively well studied. Hla expression is primarily regulated by the quorum-sensing accessory gene regulator (Agr) system, which is usually present from late logarithmic growth to quiescence (Singh and Ray, 2014). The Agr regulatory network is activated due to the accumulation of autoinducible peptide (AIP), which further passes through RNAIII effectors to induce transcription and expression of virulence factors, such as hemolysins, enterotoxins, and proteases (Seilie and Bubeck Wardenburg, 2017; Kane et al., 2018; Salam and Quave, 2018). To this end, we first evaluated the transcription levels of several key genes in the Agr system by qPCR. The results further confirmed that the transcript levels of the upstream Hla-directly regulated genes, namely, *agrA* and *RNAIII*, were significantly reduced in echinatin-treated *S. aureus*. Subsequently, we also observed that echinatin inhibited the transcription and expression levels of Hla. In addition, *S. aureus* pvl and psmα, two essential virulence factors that are directly regulated by RNAIII and AgrA, respectively, were also decreased in a dose-dependent manner with increasing echinatin concentrations. These results suggested that echinatin, which is similar to isorhamnetin (Jiang et al., 2016) and lysionotin (Jiang et al., 2016) and lysionotin (Teng et al., 2017), may inhibit hemolysis by regulating hemolysin expression through the Agr system.

Because Hla secreted by *S. aureus* is closely associated with the pneumonia infection model, we evaluated the efficacy of echinatin *in vivo* in a pneumonia model. Echinatin has been shown to spread rapidly, reaching Cmax and longer T1/2 quickly (Li et al., 2019). However, the oral bioavailability of echinatin is poor (~6.81%), while the treatment of mice infected with *S. aureus* pneumonia in this study was administered subcutaneously, which avoids the problem of the first-pass effect to some extent. In the *in vivo* infection model, echinatin increased the survival of mice with MRSA-induced pneumonia and reduced signs of infection, such as the extent of inflammation in lung tissue, extent of edema, and lung bacterial load. MRSA infection triggers a complex host response cascade in the lungs that typically results in microvascular damage and accumulation of fluid and immune cells that impair respiratory function (Enkhbaatar et al., 2008). Consistent with our findings, flow cytometric experiments revealed that treatment with echinatin reduced inflammatory damage in the lungs of mice with a significant reduction in the number of neutrophils and macrophages compared to the infected group. In summary, the present results confirmed the significant therapeutic effect of echinatin against *S. aureus*-induced pneumonia in mice.

Notably, we performed pan assay interference structures (PAINS) motif identification based on the SwissADME method, and the results showed that echinatin did not contain PAINS motifs, which excluded the possibility of generating false-positives.

In conclusion, the present study showed that echinatin is a promising inhibitor of *S. aureus* virulence. Echinatin was highly efficient in depriving *S. aureus* of virulence factors, thereby suggesting that echinatin is a new candidate compound for the fight against MRSA.

## Data availability statement

The raw data supporting the conclusions of this article will be made available by the authors, without undue reservation.

## Ethics statement

The animal study was reviewed and approved by the Institutional Animal Care and Use Committee (IACUC) of Jilin University.

## Author contributions

MY and YZ conceived and designed the experiments. WZ, QG, ZT, XM, and ZW performed and analyzed the experiments. WZ, JG, and LW revised the manuscript. All authors contributed to the article and approved the submitted version.

## Conflict of interest

The authors declare that the research was conducted in the absence of any commercial or financial relationships that could be construed as a potential conflict of interest.

## Publisher’s note

All claims expressed in this article are solely those of the authors and do not necessarily represent those of their affiliated organizations, or those of the publisher, the editors and the reviewers. Any product that may be evaluated in this article, or claim that may be made by its manufacturer, is not guaranteed or endorsed by the publisher.
