# Supplementary material for: The natural product, echinatin, protects mice from methicillin-resistant Staphylococcus aureus pneumonia by inhibition of alpha-hemolysin expression
Source: Front Microbiol. 2023 Apr 14;14:1128144. doi: 10.3389/fmicb.2023.1128144 (PMC10140358; doi:10.3389/fmicb.2023.1128144)
Supplement: Supplementary file 1 [file Data_Sheet_1.PDF]

Supplementary Material

The Natural Product, Echinatin, Protects Mice from Methicillin-resistant *Staphylococcus aureus* Pneumonia by Inhibition of Alpha-hemolysin Expression

Wei Zhang, Qing Gong, Zhitong Tang, Xin Ma, Zhuoer Wang, Jiyu Guan, Li Wang, Yicheng Zhao, Ming Yan*

*Correspondence

Ming Yan, mingyan.ccucm@outlook.com

Table of contents

Figure .1 Quality test report of echinatin.

Figure .2 Analysis of pulmonary immune cell proportions in mice with *S. aureus* pneumonia by flow cytometry.

Table .1 Screening of effective inhibitors from small molecule libraries by hemolysis assay.

Figure .1 Quality test report of echinatin.

成都普菲德生物技术有限公司

Chengdu Pufei De Biotech Co., Ltd

Tel:86-28-82610909 Fax:86-28-81711798

Email:sc-victory@163.com 地址：成都市温江区青啤大道 319 号

刺甘草查尔酮

Retrochalcone/Echinatin

检测信息（Detection Information）	色谱柱（Column）：Kromasil 100-5-C18 4.6*250mm<br>流动相(Mobile phase): A Acetonitrile B 0.1% phosphoric acid solution<br>0min38%A，15min38%A，25min80%A<br>检测波长（Detection mode）：360nm<br>柱温（Column temperature）：30℃

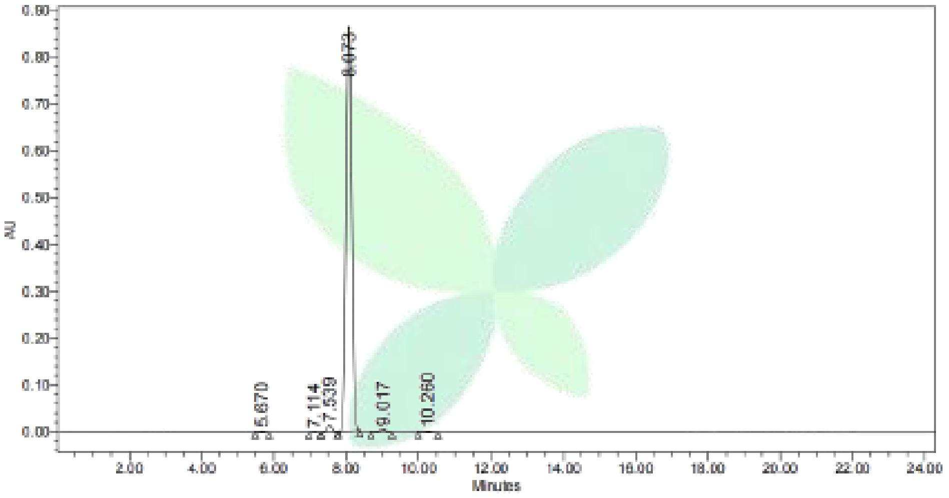

	RT	Area	Height	% Area
1	5.670	15903	1988	0.17
2	7.114	5169	587	0.05
3	7.539	135520	13597	1.44
4	8.073	9184182	864721	97.43
5	9.017	65004	5358	0.69
6	10.260	20410	1658	0.22

Figure. 2 Analysis of pulmonary immune cell proportions in mice with *S. aureus* pneumonia by flow cytometry.

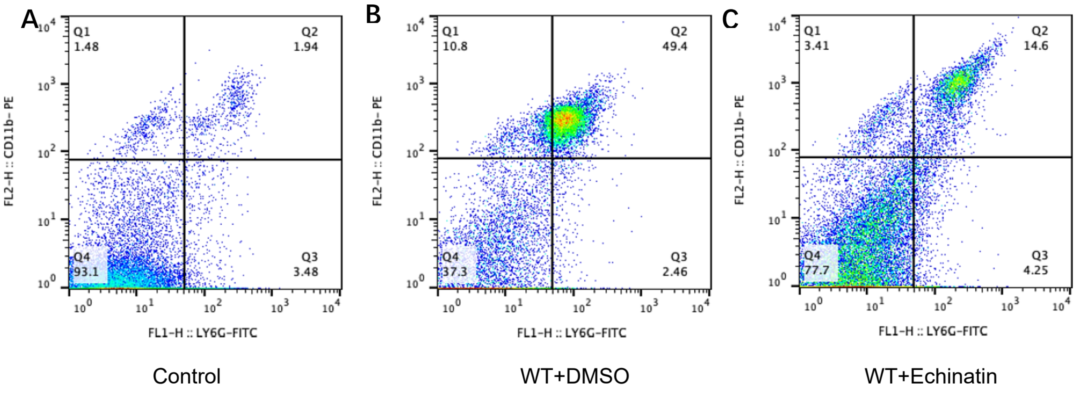

Figure. 2 Analysis of pulmonary immune cell proportions in mice with *S. aureus* USA300 pneumonia by flow cytometry. (A) Proportions of neutrophils and macrophages in the lungs of untreated mice. After 24 hours of infection with *S. aureus* USA300, mice were euthanized, and lung tissue cells were collected to detect the proportions of neutrophils and macrophages in the lungs of the (B) infected group and (C) echinatin-treated group.

Table. 1 Screening of effective inhibitors from small molecule libraries by hemolysis assay

No.	Compounds	CAS	Categories	Inhibition effect
1	Bilobeti	521-32-4	Biflavones	—
2	Irigenin	548-76-5	Isoflavones	—
3	Baohuoside V	118544-18-6	Flavonols	—
4	Baohuoside I	113558-15-9	Flavonols	—
5	Baohuoside VII	119730-89-1	Flavonols	—
6	Procyanidin C1	37064-30-5	Biflavones	—
7	Apiin	26544-34-3	Flavones	—
8	Alpha-Naphthoflavone	604-59-1	Flavones	—
9	*Sinensetin*	2306-27-6	Flavones	—
10	Schaftoside	51938-32-0	Flavones	—
11	Galangin 3-methyl ethe	6665-74-3	Flavonols	—
12	Flavokawain B	1775-97-9	Chalcones	—
13	7,4'-Dihydroxyflavone	2196-14-7	Flavones	—
14	Taxifolin	111003-33-9	Flavanonols	—
15	Prunetin	552-59-0	Isoflavones	—
16	Leucoside	27661-51-4	Flavonols	—
17	Engeletin	572-31-6	Flavanonols	—
18	Mirificin	103654-50-8	Isoflavones	—
19	Artemitin	479-90-3	Flavonols	—
20	Complanatuside	116183-66-5	Flavonols	—
21	Complanatoside A	146501-37-3	Flavonols	—
22	Tectoridin	611-40-5	Isoflavones	—
23	Trifoside	154-36-9	Isoflavones	—
24	Typhaneoside	104472-68-6	Flavonols	—
25	Narirutin	14259-46-2	Flavonones	—
26	Isomangiferin	24699-16-9	Polyphenols	—
27	Ieariline	489-32-7	Flavonols	—
28	Orientin	28608-75-5	Flavones	—
29	Homoorientin	4261-42-1	Flavones	—

30	Trihydroxyethylrutin	7085-55-4	Flavones	—
31	Isoschaftoside	52012-29-0	Flavones	—
32	Trifolirhizin	6807-83-6	Flavonoids	+
33	Desmethylglycitein	17817-31-1	Isoflavones	—
34	Luteolin-3-O-beta-D-glucuronide	53527-42-7	Flavones	—
35	Ginkgetin	481-46-9	Biflavones	—
36	Tilianin	4291-60-5	Flavones	—
37	Kuwanon G	75629-19-5	Flavones	—
38	Echinatin	34221-41-5	Chalcones	+++
39	Spinosin	72063-39-9	Flavones	—
40	Vicenin 2	23666-13-9	Flavones	—
41	Pectolinarin	28978-02-1	Flavones	—
42	Oroxin B	114482-86-9	Flavones	—
43	Oroxin A	57396-78-8	Flavones	—
44	Isobacachalcone	20784-50-3	Chalcones	+++
45	4',7-Dimethoxy-5-Hydroxyflavone	5128-44-9	Flavones	—
46	7,4'-Dihydroxyflavone	2196-14-7	Flavones	—
47	4',5-Dihydroxyflavone	6665-67-4	Flavones	—
48	Neoisoliquiritin	59122-93-9	Chalcones	—
49	Neoeriocitrin	13241-32-2	Flavonones	—
50	Hesperetin 7-rutinoside	520-26-3	Flavonones	—
51	Pedalitin permethyl ether	2306-27-6	Flavones	—
52	Vincetoxicoside B	22007-72-3	Flavonols	—
53	Eriocitrin	13463-28-0	Flavonones	—
54	Wogonoside methyl ester	82475-01-2	Flavones	—
55	Wogonoside	51059-44-0	Flavones	—

Triton and USA300 were used as negative controls for hemolytic activity, and PBS and myricetin (Wang et al., 2020) were used as negative controls. "—" represents no hemolysis inhibition effect; "++" represents significant inhibition of hemolysis; "+++" stands for extremely significant inhibition of hemolysis.

References

Wang, T., Zhang, P., Lv, H., Deng, X., and Wang, J. (2020). A Natural Dietary Flavone Myricetin as an α-Hemolysin Inhibitor for Controlling Staphylococcus aureus Infection. *Front Cell Infect Microbiol* 10**,** 330. doi: 10.3389/fcimb.2020.00330.
